# Clinical Lecture on the Treatment of Acute Rheumatism, Delivered at St. Mary’s Hospital, London

**Published:** 1863-04

**Authors:** Thos. K. Chambers


### SELECTED.

### CLINICAL LECTURE ON THE TREATMENT OF ACUTE RHEUMATISM,

### DELIVERED AT ST. MARY'S HOSPITAL, LONDON.

By THOS. K. CHAMBERS, M.D.

*Gentlemen*—Rheumatic Fever is a pleasant disease—I mean for the doctor to treat, though not for the patient to bear. It is pleasant for him to treat it, because he then feels himself strong and useful. In the first place, he can by the judicious exercise of his art, insure the sufferers against several perils to which the nature of their complaint normally subjects them. Again, he can save them much pain. Thirdly, he can shorten the normal duration both of the illness and of the convalescence. Truly, in most diseases he can effect one or the other of these objects, but in none, I think, so many of them, so surely or so simply as in rheumatic fever.

Rheumatic fever is also a pleasant disease to lecture about. It presents a simple uniform type, so that the short descriptions you have had in the systematic course on the Practice of Medicine are found really applicable at the bedside, without the necessity of guarding them with all sorts of exceptions and variations, which clinical teachers are so often obliged to resort to in other cases. And a very simple uniform treatment may be recommended, which hardly ever (if ever) requires modification. So that if your authority with your patient is sufficient, and you are certain of your diagnosis, you never need to call in the assistance of a physician.

You see taken in the wards almost weekly specimens of the mode of treatment I adopt. My present business is to tell you the reasons for it.

1. The patients are bedded in a peculiar fashion. All linen is strictly forbidden to touch the skin. A slight calico shirt or shift may be allowed; but if they possess under clothing only of the prohibited sort, they are better naked. Sheets are removed, and the body carefully wrapped in blankets, which are so arranged as to shut off all accidental draughts from the head. The newest and fluffiests blankets that can be got are used. The bedclothes being put so are kept so, and students are warned that, when they listen to the sound of the heart, they must not throw open the blankets, but insert their stethoscope (first warmed) between the folds.

2. These joints or limbs which are swollen, red, or painful, are wrapped up in flannels soaked with a hot fomentation consisting of decoction of poppy heads, with half an ounce of carbonate of soda to each pint.

3. The following drugs are prescribed with a curative intention:

(*a*) If the skin is red, swollen, and painful, about the joints —if the cellular tissue around the muscles is infiltrated and sensitive, so that motion is impossible or exquisitely painful—more especially if these phenomena are metastatic, leaving one part free and attacking another,—then they get the alkaline treatment pure and simple; they have a scruple of bicarbonate of potash in camphor water every other hour night and day when awake.

(*b*) If the above named phenomena are insignificant, and the pain is felt more in the bones—if it is intensified rather by pressure than by motion—if it is fixed in one spot and not metastatic,—then I add two grains of iodide of potassium to each dose; and directly the symptoms have taken a turn towards alleviation, I leave off the alkali altogether, and gave only the iodide.

4. Opium, as a palliative, is given in exact proportion to the degree of subjective sensation of pain. If one grain be not enough to entice sleep, a grain and a half is administered; if that do not avail, two grains. Directly the pain is better, the quantity of the drug is diminished. Nothing effects the desired object so much as pure opium.

5. If the pain remains fixed in one point, instead of leaving it like in other places, leeches are applied there, and the part is kept poulticed. When we can get them, young laurel leaves, bruised, are mixed with the poultice.

6. The latter statement is applied also to the cardiac region, if the heart has become inflamed either inside or out. The pain is taken as an indication of the extent to which the leeching is to be pushed, so soon as it is proved by auscultation that such pain arises from inflammation of the heart, and not from rheumatism of the pectoral muscles. The constant application of the poultice is made imperative.

7. The diet is varied in some degree according to the antecedent circumstances of the patients. If they have been robust healthy persons before the attack they will bear a good deal of starvation, and they are put on our "simple diet"—to wit, bread and butter, gruel, and tea, in quantities practically at discretion. If previously they have been ill nourished, by reason of either ill health or poverty, a pint of broth or beef-tea is added.

I will now proceed to comment on the several items of treatment.

1. It is impossible to exaggerate the importance of extreme repose and an even high temperature to the skin in rheumatic fever. It is worth all the other means of relief put together. Since I have instructed my nurses to adopt it in every instance during the last eight or nine years, I have had pericarditis come on in only one patient previously sound, and that was a girl who was taking mercury and opium, and I suspect had exposed her chest a good deal to the air.

The *rationale* of this is very simple. Rheumatic inflammation is an injury to nutrition which is entirely compensated for the restored function. It passes away and leaves no after-sign, no wound, no scar. This is what happens if the part affected is kept perfectly still. But should duties be required of it, which it is unequal to perform in its imperfect condition—should necessity or ignorance lead the patient to keep moving a swollen joint, for example—then common inflammation is superadded. Then the pain and swelling become fixed, and no metastasis can take place. You see this frequently in the poor working people, who, through ignorance of consequences, strive to go on with their employment to the latest minute. Laborers come into the hospital with the disorder fixed in their knees, carpenters in their elbows, laundresses in their wrists; so that you may make a shrewd guess at their trade from the part where the disorganizing inflammation is situated. Pain may be called by excellence *the* proof of beneficent design in God's laws as shown in disease as a warning to abstain from that which excites it. The pain of rheumatism is a call to voluntary absolute rest. Now in the joints this is easily obtained, and under any treatment you never see a joint become affected with disorganizing inflammation after a patient has once taken to his bed. But there is one organ whose business admits of no repose; the heart *must* keep beating, at whatever cost; and the heart accordingly is well known to be fatally apt to be struck with common fibrinous inflammation at all stages of the disease. Taking a lesson from what I

have noticed in the joints, I try and assist the heart to gain, not, of course, the Utopia of absolute rest, but the nearest approach that is possible.

Perhaps you may think that object would be attained by simply confinement in bed and the horizontal posture. But it is not so. Next to jumping and running there is nothing gives the heart more work to do as change of temperature. Let the physiologist observe the healthy organ, and the physician examine it in a state of disease, and they will find that the addition or subtraction of heat to the surface of the body is accompanied by a longer and stronger stroke as felt by the finger, by a longer and stronger sound as heard by the ear in the cardiac region. What is technically called "the interval" is shortened; and thus is encroached upon the only wink of sleep the industrious muscle ever indulges in. What does the accoucheur do who wishes to apply the strongest vivifier to the dormant circulation of a still-born baby? He dashes cold water and cold air on the skin; he rubs the chest dry, and applies hot cloths; again he dashes it with cold, making as many changes of temperature as he can. What the accoucheur is so anxious to accomplish, we are most anxious to avoid; and I feel sure that it is the consequence of guarding patients with rheumatic fever from the influence which variations have over the dependencies of the pneumogastric nerve that the treatment now advised is so successful. I never have pericarditis come on when it is once fairly begun.

I scarcely need to say that the most important part as respects the attainment of the accoucheur's object and our opposite object is the chest and neck. He applies his stimulus especially there, and there we must as carefully watch against it. As a student I used to see many and many a case of pericarditis brought on by the careless way in which the chest was exposed in the daily stethoscopic examination. It is necessary, of course, to listen to the heart thus frequently, in order to convince yourselves of the absence of morbid sounds under the plan I am advising; but by warming the stethoscope in your pocket or under your axilla, and making the blanket into a tube by which to insert it, you put the patient to a minimum degree of danger.

You saw a fortnight ago an instance of the danger of the exposure I have been deprecating. Margaret K———, aged twenty-three, was admitted March 28th, for rheumatic fever in the arms and legs; from this she recovered perfectly without any affection of the heart, and was transferred to the Convalescent Ward.

On April 17th she had a relapse, principally affecting the legs, and on the 19th I found her in bed again. By an oversight she had not been blanketed, and when I felt the cold sheets damp with the patient's perspiration, I was not sur prised that she complained of constriction across the chest. You heard me rebuke the nurse in no measured terms, and prognosticate evil. With justice; for before two days were over, there was a melancholy systolic murmur distinctly audible. I trust this case has been a warning to you.

2. By comparing occasional cases one limb wrapped in fomentations of simple hot water with another where decoction of poppy heads were used, I come to the conclusion that either the viscid vegetable matter or the small quantity of opium in the poppy heads contributed towards alleviating the pain a little. And a similar experiment has led me to the same opinion as respects an alkaline carbonate.

3. With unimportant exceptions I have treated every patient on the alkaline plan for the last seven years, being convinced of its power to shorten and alleviate the disease by the statistical deductions of Dr. Garrod. In a great majority of the cases very rapid relief begins with the commencement of the treatment, and continues permanent. But in a certain number no effect appears to be produced, sometimes even after the urine had been made alkaline. In a few of these there is apparently committed a pardonable error of diagnosis, and the patient is gouty. In a few also we are deceived by gonorrhœal rheumatism—a disease allied to pyæmia, and requiring quite different management. Still there are a certain number of instances where true rheumatic inflammation is very obstinate, and does not yield to the alkaline method. And in these you will find the periosteum and perichondium affected. When, then, after five or six days the patient is better, or but little better, I add as I told you, iodide of potassium to the potash, and in a few days more continue it alone during the convalescence. And, of course, if I am enambled to make this condition of periosteum out of the first visit, I begin such treatment straightway.

I mentioned just now that I had in a few instances, for exceptional reasons, not given the alkaline treatment for rheumatic fever. Amongst these are included a middle-aged laborer and his wife, both attacked together, and just recovered, in which cases you saw no drugs given during the acute stage. The object of this omission was partly to disabuse my own mind of a suspicion that the alkalies might cause, or augment the anæmia and weakness so general in the convalescence of rheumatic fever; or, perhaps, might give rise to relapses by interrupting the course of the disease. You saw that the loss of flesh and strength was in these two cases as great as usual, if not greater than in the majority of the examples exhibited to you in the wards, satisfying us that it is the disease, not the remedy, which is to blame for it. You saw, also, that one of the patients (the man) had a relapse, showing that to nature, and not to art, is to be attributed this unfortunate occurrence so frequent in rheumatic cases.

Partly, also, I omitted drugs to remind you that you do not carry in your medicine chests absolute powers—that rheumatic fever is a state in which the forces of life move in a circle, in a road which leads of itself back towards health—not a chronic disorganizing process, whose path may be described as a straight line, approaching nearer and nearer to death the farther it goes. It ends of its own accord, or at all events without the aid of drugs, often in a few days, often (as you saw here) in a time quite as short as could have been expected had medicine been administered. This consideration is needful to enable you to estimate properly the value of numerical arguments and to understand that a very large collection of cases, much larger than your experience probably will ever supply to you, is required to prove the ability of a drug to shorten rheumatic fever. If you forget this, you risk being misled by a fallacy, at an instance of which, applied to this very disease, I was amused a few years ago. I had an interview with an irregular practitioner, (very irregular iudeed,) who told me that he gave no medicines, but followed "the method of St. James;" he "anointed with oil those that were sick, and the Lord raised them up." As evidence of the success of his plan, he gave me the history of two attacks he had experienced of rheumatic fever. In the first he was treated *secundem artem*, and was laid up for more than three weeks; in the second, he obeyed the perversion of Scripture above quoted, and was out of bed in five days. Of course he was perfectly impervious to argument.

Do not misunderstand my words, as if I intended to be sceptical of the proof adduced by Dr. Garrod of the success of the alkaline treatment in shortening the average duration of our patient's pains. I think he fairly proves his point by the numerical method. But besides that, the use of such drugs is quite in harmony with the principles of restorative medicine. The deficiency of the alkali in the body is shown in all quarters by the appearance of free acids. In indubitable cases left without treatment, the sweat is acid; the saliva is acid; the urine, instead of being normally acid, is intensely acid; the breath even smells acid. The blood, indeed, remains alkaline, fortunately for the life of the patient; but that is only done at the expense of becoming exceedingly watery, and inducing the anæmia which is so characteristic of the convalescence of rheumatic fever. If the blood is aqueous, and contains less solids than normal at the same time that the salts bear their usual proportion to the rest of the solids, it is obvious that there must be a great deficiency of these salts in the body. Though the blood therefore be not acid, it is easy to understand that it carries less alkali than it ought to do.

A real deficiency, then, is attempted to be restored by the alkaline treatment. And when we think what a great mass of living matter it is over the whole of which this blatant deficiency exists, then is explained the necessity found for large and repeated doses, which all good observers insist upon. To give a few grains three times a day is merely playing at healing, and cannot be reckoned as treatment at all. I do not think anything less than half an ounce in the twenty-four hours of the bicarbonate of potash is of use. If this runs off straight by the kidneys, making the urine alkaline too rapidly, it is of little avail; but if it mixes with the mass of the corporeal fluids, and is some time before it affects the reaction of the renal secretion, the advantage is sensibly appreciated by both the patient and his attendants.

The employment of iodide of potassium is purely empirical. By none can the fact be explained that this remarkable substance restores their normal functions to several tissues—most notably to those sparingly supplied with bloodvessels, such as cartilaginous and white, hard, fibrous parts, the periosteum, the sheaths of tendons and of nerves, the hair, the nails, and the outer layers of skin. On these grounds it is employed when rheumatism and even when gout attacks the tendinous and internal tegumentary parts of the joints and limbs. And I think one cannot doubt the assurances of the sufferers that they feel better for it, however inexplicable the fact may be.

4. Opium is administered purely as anæsthetic. There is no reason to think it either shortens or lengthens the duration of the disease. Curiously enough, it does not usually produce constipation so long as the painful condition which it is given to alleviate remains. Should, however, that result follow, the inconvenience is easily obviated by adding two or three grains of good extract of colocynth to the opium pill.

5. The treatment by leeches and poultices of the common inflammation which has supervened on the rheumatic in joints over-exerted during their weak state, has nothing special about it. It will usually prevent disorganization, because in point of fact the inflammation is very slight and diffused.

I have told you inflammation of the heart does not come on in patients who have once been placed and kept under the treatment detailed to you; but in a good many the exposure they have been subject to previously, and sometimes, perhaps, the necessary time spent in the waiting-room before admission, gives you, unfortunately, the opportunity of seeing this complication treated. I feel satisfied that it need make no difference in the applicability of the alkaline method; indeed, it rather determines me to insist on its being fully carried out; it determines me also to be more than usually careful about the maintenance of temperature by blankets, and to direct this attention to the chest in special by the retention of continuous poultices on the cardiac region. From six to twelve leeches are applied immediately. These usually relieve the pain somewhat; but if it returns again next day, they are freely repeated again and again. The pain is the best indication of the acuteness of inflammation in serous membranes, and as long as acute inflammation lasts, leeches and poultices are the best remedies for it. To mercury I have never been able to trace any advantage at this stage; indeed, I am not sure that it does not dispose to pericarditis by increasing the proportion of fibrin to the other constituents of the blood. Perhaps after effusion has taken place it may be useful; but I am not quite satisfied that it is desirable in all cases even then. Opium may be given in full doses, and far from being contra-indicated in cardiac inflammation, is all the more urgently demanded; for it certainly does control and lower the hurry of circulation, which is so dangerous. Under its use the pulse is diminished in frequency, sometimes even below the normal standard; and this must surely be an important object in a state induced by the continuous motion of the organ.

The treatment of pericarditis admits of no delay. Lost minutes are more hurtful here than in any disease I know of. Send for leeches, and have them applied immediately, our suspicions are aroused by an abnormal murmur; and if they are not at hand, cup the cardiac region. It is better even to anticipate evil than to be too late. On this principle you saw me a fortnight ago leech and poultice the heart of the young woman before mentioned (case of Margaret K———), where you could detect no friction in the pericardium, and you wondered at my "sharp practice;" but the fact is, it was a case of relapse during convalescence, and as the patient was in the convalescent ward, the nurse negligently omitted to put her in blankets; the cold, damp linen was beginning to do its work, and the lengthening heavy systole of the left ventricle, accompanied by a sense of constriction, and pain on pressure, warned me to try and prevent the threatened inflammation. I was only partially successful; for the anticipated evil did come as predicted, and in two days' time an exo-cardial murmur was distinct enough, though I am convinced it was in a much milder form than would have happened without the leeches.

7. In rheumatic fever there is a painful necessity for restricting the supply of nutriment. If animal food be given, it appears to turn into lactic acid, or at all events to increase the quantity of animal acid in the body. Even when the pains are gone, and there is such an urgent necessity for replacing the lost flesh, animal food will sometimes bring on a relapse. Hence in rheumatic fever, alone, perhaps, of all diseases, I give the patient less food than their inclinations dispose them to take. Meat especially seems to disagree, and you must very cautiously get back to "ordinary diet" after rheumatic fever, or you run the risk of losing more by a second attack of the disease than is gained by haste. Vegetable food does not throw them into the same danger, and thus by dint of rice pudding, porridge, gruel, bread, mashed potatoes, and so on, you can generally succeed in stopping the mouths which are often so loudly complaining of starvation. If you cannot succeed in staying the appetite by this persuasion, I fear it is your duty to be cruel, for observation will soon convince you of the dangerous effects of animal food.—*London Lancet.*

